# Interplay between chronic inflammation and clonal haematopoiesis of indeterminate potential in Behçet’s disease

**DOI:** 10.1186/s13075-023-03014-w

**Published:** 2023-03-02

**Authors:** Jihye Park, Hongyul An, Jiwoo Lim, I Seul Park, Mi Hyun Kim, Ji Hyung Kim, Seung Won Kim, Young Il Koh, Eun Young Lee, Jae Hee Cheon

**Affiliations:** 1 Department of Internal Medicine, Severance Hospital, Yonsei University College of Medicine, 50-1 Yonsei-Ro, Seodaemun-Gu, Seoul, 03722 South Korea; 2 Institute of Gastroenterology, Severance Hospital, Yonsei University College of Medicine, Seoul, South Korea; 3 Genome Opinion Inc, Seoul, South Korea; 4 Brain Korea 21 PLUS Project for Medical Science, Yonsei University College of Medicine, Seoul, South Korea; 5 Division of Rheumatology, Department of Internal Medicine, Seoul National University College of Medicine, Seoul, South Korea; 6 Division of Hematology and Oncology, Department of Internal Medicine, Seoul National University Hospital, Seoul, South Korea; 7 Division of Rheumatology, Department of Internal Medicine, Seoul National University Hospital, 101 Daehak-Ro, Jongno-Gu, Seoul, 03080 South Korea

**Keywords:** Behçet’s disease, Clonal haematopoiesis of indeterminate potential, Inflammation

## Abstract

**Background:**

Clonal haematopoiesis of indeterminate potential (CHIP) is a predisposition to haematological malignancy whose relationship with chronic inflammatory diseases, such as cardiovascular diseases, has been highlighted. Here, we aimed to investigate the CHIP emergence rate and its association with inflammatory markers in Behçet’s disease (BD).

**Methods:**

We performed targeted next-generation sequencing to detect the presence of CHIP using peripheral blood cells from 117 BD patients and 5004 healthy controls between March 2009 and September 2021 and analysed the association between CHIP and inflammatory markers.

**Results:**

CHIP was detected in 13.9% of patients in the control group and 11.1% of patients in the BD group, indicating no significant intergroup difference. Among the BD patients of our cohort, five variants (*DNMT3A*, *TET2*, *ASXL1*, *STAG2*, and *IDH2*) were detected. *DNMT3A* mutations were the most common, followed by *TET2* mutations. CHIP carriers with BD had a higher serum platelet count, erythrocyte sedimentation rate, and C-reactive protein level; older age; and lower serum albumin level at diagnosis than non-CHIP carriers with BD. However, the significant association between inflammatory markers and CHIP disappeared after the adjustment for various variables, including age. Moreover, CHIP was not an independent risk factor for poor clinical outcomes in patients with BD.

**Conclusions:**

Although BD patients did not have higher CHIP emergence rates than the general population, older age and degree of inflammation in BD were associated with CHIP emergence.

## Background

Clonal haematopoiesis of indeterminate potential (CHIP), defined as the presence of somatic mutations and clonal expansion in haematopoietic stem cells of the blood or bone marrow, is a common ageing-associated condition that begins in middle age [1]. CHIP, a variant allele frequency (VAF) exceeding 2%, is not a malignancy but rather a predisposition to haematological malignancy and cardiovascular disease [2, 3]. Furthermore, CHIP could contribute to chronic inflammation; for example, loss of ten eleven translocation-2 (*TET2*) upregulated inflammatory mediators, such as interleukin-6, in mouse colitis and mouse atherosclerosis models [4, 5].

In contrast, inflammation from pre-existing comorbidities, including autoimmune diseases or infections, can cause mutagenesis via DNA damage and promote CHIP [6]. CHIP was reportedly identified in 17% of rheumatoid arthritis patients and 30.4% of patients with anti-neutrophil cytoplasmic antibody (ANCA)–associated vasculitis [7, 8]. In patients with ulcerative colitis (UC), Zhang et al. identified that 12.8% had CHIP variants and interpreted that patients with UC may show a slightly higher tendency toward a CHIP appearance after the sixth decade compared to the general population [9]. Subpopulations of CHIP with *DNMT3A* mutations were significantly associated with higher serum interferon-gamma levels [9]. In patients with inflammatory bowel disease co-occurring with haematologic disease, Cumbo et al. identified 85% of CHIP variants and suggested a biological link between the proinflammatory environment and haematologic malignancy onset [10].

Behçet’s disease (BD) is a chronic inflammatory immune-mediated disorder that causes recurrent oral and genital ulcers and skin, joint, eye, neurological, and gastrointestinal involvement [11, 12]. Patients with BD have a higher risk of haematological malignancy (pooled relative risk, 2.58; 95% confidence interval [CI], 1.61–3.55) [13, 14]. However, no studies have investigated the relationship between CHIP and BD. Therefore, here, we aimed to characterise the occurrence of CHIP in patients with BD and analyse the associations between CHIP and inflammatory markers in patients with BD and their outcomes.

## Methods

### Study design and population

For the control group, we collected blood samples from participants during a routine health check-up for screening purposes at the Seoul National University Hospital Healthcare System Gangnam Center between January 2014 and January 2017 (no. 1980–121-1056). For the BD group, we collected blood samples from patients with BD during routine outpatient visits from two tertiary hospitals (Severance Hospital in Seoul, Korea and Seoul National University Hospital in Seoul, South Korea) between March 2009 and September 2021. Systemic BD was diagnosed based on the International Criteria for Behçet’s Disease [15]. Patients with intestinal BD classified as having definite, probable, or suspected intestinal BD were enrolled in this study based on established diagnostic criteria [16]. This study was conducted in accordance with the ethical guidelines of the Declaration of Helsinki. Informed consent was obtained from all individuals, and the study protocol was approved by the Institutional Review Board of Seoul National University (no. 1606–116-772) and Severance Hospital (no. 2012–0039-030).

### Targeted gene sequencing and variant annotation

Genomic DNA was extracted from the peripheral blood. Targeted next-generation sequencing was performed using a custom panel consisting of 89 *CHIP* genes frequently involved in *CHIP*, *DNMT3A*, *TET2*, *ASXL1*, *JAK2*, and *TP53*, with an average target depth of over 1000 × . All non-synonymous variants with a variant allele frequency of 1.5–30% were considered CHIP variants. Common germline variants listed in the gnomAD,10–0 Genomes v3, ESP6500, and ExAC databases and an internal panel of 1000 Koreans were excluded.

### Data collection and study outcomes

Demographic and clinical information was collected at outpatient visits, including age, disease duration, sex, body mass index, smoking history, presence of intestinal BD, previous and current medications, and laboratory findings. Medical accelerations for BD, including immunomodulators and biologic agents, surgical requirements for BD, BD-related hospitalisation, and BD-related emergency room visits, were also investigated to determine the patients’ long-term outcomes.

### Statistical analysis

Continuous data are expressed as mean and standard deviation (mean ± SD) or median and range, while categorical variables are expressed as proportion (%). An independent *t*-test (or Mann–Whitney *U* test) was used to compare the continuous variables, while the *Χ*^2^ test (or Fisher’s exact test) was used for categorical variables as appropriate. Multivariate logistic regression analyses were performed to identify the independent risk factors for CHIP after the adjustment for various confounders. All statistical analyses were assessed with the Statistical Package for Social Sciences (SPSS version 24.0; IBM Corp., Armonk, NY, USA). *p* values < 0.05 were considered statistically significant.

## Results

### Association between CHIP and BD development

**Fig. 1:**
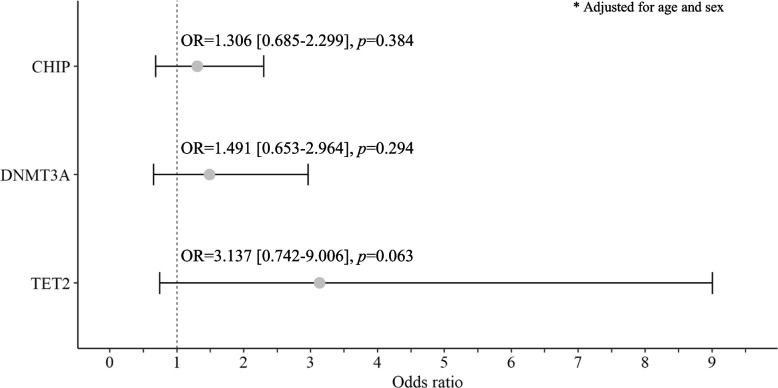
Forest plot of adjusted odds ratios for the incidence of Behçet’s disease adjusted for age and sex. CHIP, clonal haematopoiesis of indeterminate potential; OR, odds ratio

Blood samples were collected from 5004 healthy controls (control group) and 117 BD patients (BD group). We identified CHIP in 13.9% of the control group and 11.1% of the BD group, indicating that there was no significant difference between the two groups (Fig. 1). In patients with BD, five variants (*DNMT3A*, *TET2*, *ASXL1*, *STAG2*, and *IDH2*) were detected in our cohort, and *DNMT3A* mutations were the most common (7.5%), followed by *TET2* mutations (1.9%).

### CHIP and disease activity of BD

**Fig. 2:**
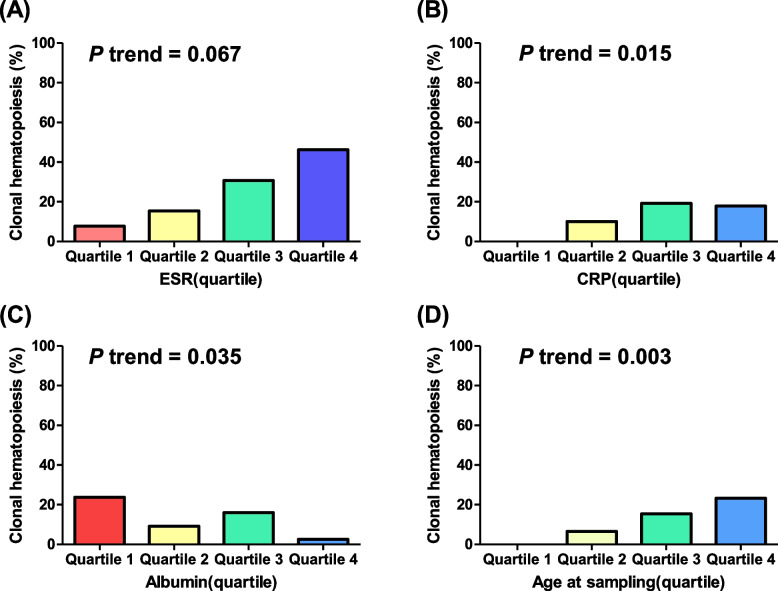
CHIP positivity rate according to **A** ESR, **B** CRP level, **C** albumin level at diagnosis, and **D** age at blood sampling. CHIP, clonal haematopoiesis of indeterminate potential; CRP, C-reactive protein; ESR, erythrocyte sedimentation rate

Patients with BD and CHIP were older and had a higher platelet count, erythrocyte sedimentation rate (ESR), and C-reactive protein (CRP) level and lower albumin level at diagnosis than BD patients without CHIP (Table 1). Patients with a higher baseline ESR showed a higher CHIP-positive tendency than those with a lower baseline ESR (4.0%, 8.0%, 13.3%, and 28.6%, respectively; *p* trend = 0.067; Fig. 2A). Patients with a higher baseline CRP level showed a significantly higher CHIP-positive rate than those with a lower baseline CRP level (0.0%, 10.0%, 19.2%, and 27.9%, respectively; *p* trend = 0.015; Fig. 2B). Patients with a higher baseline albumin level showed a significantly lower CHIP-positive rate than those with a lower baseline albumin level (23.8%, 9.4%, 16.0%, and 2.6%, respectively; *p* trend = 0.035; Fig. 2C). Older patients showed a significantly higher CHIP positivity rate than younger patients (0.0%, 6.5%, 15.4%, and 23.3%, respectively; *p* trend = 0.003; Fig. 2D).

**Table 1:** Baseline characteristics of Behcet’s disease (*N* = 117)

Variables	CHIP( +) (*n* = 13)	CHIP( −) (*n* = 104)	*p value
Age at blood sampling (years)	58.1 ± 11.4	45.1 ± 13.3	0.001
Disease duration (years)	6.6 ± 4.1	7.6 ± 5.9	0.540
Sex (male)	8 (61.5%)	48 (46.2%)	0.381
Body mass index (kg/m^2^)	21.1 ± 3.0	21.8 ± 3.4	0.507
Smoking history	4 (30.8%)	29 (27.9%)	1.000
Symptom of BD			
Oral ulcer	5 (38.5%)	57 (54.8%)	0.378
Genital ulcer	1 (7.7%)	26 (25.%)	0.263
Eye manifestation	1 (7.7%)	8 (7.7%)	1.000
Skin manifestation	1 (7.7%)	38 (36.5%)	0.058
Arthritis	4 (30.8%)	25 (24.0%)	0.734
Vascular manifestation	1 (7.7%)	3 (2.9%)	0.380
Central nervous system manifestation	0 (0.0%)	1 (1.0%)	1.000
Intestinal BD	12 (92.3%)	78 (75.0%)	0.294
Previous medication			
5-ASA	12 (92.3%)	75 (72.1%)	0.179
Corticosteroid	6 (46.2%)	71 (68.3%)	0.130
Immunomodulator	7 (53.8%)	55 (52.9%)	1.000
Anti-TNF	2 (15.4%)	20 (19.2%)	1.000
Current medication			
Immunomodulator	3 (23.1%)	28 (26.9%)	0.698
Anti-TNF	0 (0.0%)	17 (16.3%)	0.029
Laboratory findings at diagnosis			
WBC count (/µL)	8511.5 ± 3510.8	7677.3 ± 2927.6	0.345
Neutrophil count (/µL)	5601.6 ± 2586.5	5064.6 ± 2748.2	0.506
Lymphocyte count (/µL)	2024.3 ± 1108.3	1873.8 ± 832.4	0.556
Haemoglobin (g/dL)	12.0 ± 1.9	12.7 ± 1.8	0.210
Platelet count (/µL)	369,000.0 ± 176,870.9	283,000.0 ± 77,279.8	0.002
NLR ratio	3.92 ± 3.96	3.82 ± 4.78	0.939
PLR ratio	259.8 ± 220.6	205.8 ± 190.3	0.785
ESR (mm/h)	69.5 ± 42.5	36.6 ± 29.8	0.001
CRP (mg/L)	30.5 ± 40.2	12.9 ± 28.0	0.046
Albumin (g/dL)	3.9 ± 0.4	4.2 ± 0.4	0.027
Clinical outcomes			
Medical acceleration (events)	7 (53.8%)	58 (56.3%)	1.000
Surgery (events)	4 (30.8%)	19 (18.3%)	0.282
Emergency room visit (events)	5 (38.5%)	32 (30.8%)	0.546
Hospitalisation (events)	6 (46.2%)	43 (41.3%)	0.772

Variables are expressed as mean ± SD or *n* (%)

*CHIP* Clonal haematopoiesis of indeterminate potential, *ASA* Aminosalicylate, *TNF* Tumour necrosis factor, *WBC* White blood cell, *NLR*, Neutrophil to lymphocyte ratio, *PLR* Platelet to lymphocyte ratio, *ESR*, Erythrocyte sedimentation rate, *CRP*, C-reactive protein

^*^p value for comparing the CHIP( +) and CHIP( −) groups

A univariate logistic analysis was performed to determine independent factors associated with CHIP in patients with BD. Older age at blood sampling (odds ratio [OR], 1.090; 95% CI, 1.030–1.153; *p* = 0.003), high platelet count at diagnosis (OR, 1.000; 95% CI, 1.000–1.000; *p* = 0.012), and high ESR at diagnosis (OR, 1.027; 95% CI, 1.009–1.046; *p* = 0.004) were positively associated with CHIP (Table 2). A high albumin level (OR, 0.228; 95% CI, 0.058–0.887;* p* = 0.033) was negatively associated with CHIP (Table 2). Age at blood sampling was the only independent factor for CHIP after multivariate logistic analysis including various confounders (OR, 1.099; 95% CI, 1.020–1.184;* p* = 0.013) (Table 2).

**Table 2:** Logistic regression analysis for CHIP (*N* = 117)

Variables	Univariate analysis			Multivariate analysis		
	OR	95% CI	*p value	OR	95% CI	*p value
Age at blood sampling (years)	1.090	1.030–1.153	0.003	1.099	1.020–1.184	0.013
Disease duration (years)	0.964	0.856–1.084	0.538			
Sex (male)	0.536	0.164–1.747	0.301			
Body mass index (kg/m^2^)	0.934	0.765–1.141	0.504			
Smoking history	0.914	0.253–3.296	0.890			
Intestinal BD	4.000	0.496–32.266	0.193			
Previous medication						
5-ASA	4.640	0.577–37.309	0.149			
Corticosteroid	0.398	0.124–1.278	0.122			
Immunomodulator	1.018	0.320–3.239	0.976			
Anti-TNF	0.745	0.153–3.633	0.716			
Current medication						
Immunomodulator	0.643	0.131–3.162	0.587			
Anti-TNF	1.000	(NS)				
Laboratory findings at diagnosis						
WBC count (/µL)	1.000	1.000–1.000	0.344			
Neutrophil count (/µL)	1.000	1.000–1.000	0.503			
Lymphocyte count (/µL)	1.000	1.000–1.000	0.553			
Haemoglobin (g/dL)	0.808	0.579–1.128	0.210			
Platelet count (/µL)	1.000	1.000–1.000	0.012	1.000	1.000–1.000	0.058
NLR ratio	1.005	0.891–1.133	0.938			
PLR ratio	1.000	1.000–1.000	0.817			
ESR (mm/h)	1.027	1.009–1.046	0.004	1.007	0.982–1.033	0.582
CRP (mg/L)	1.013	0.999–1.028	0.068			
Albumin (g/dL)	0.228	0.058–0.887	0.033	0.699	0.075–6.478	0.752

Variables are expressed as mean ± SD or *n* (%)

*CHIP* Clonal haematopoiesis of indeterminate potential, *OR* Odds ratio, *CI* Confidence interval

^*^p value for comparing the CHIP( +) and CHIP( −) groups

### CHIP and long-term clinical outcomes of BD

The development of CHIP in patients with BD was not associated with medical acceleration, surgical requirement, BD-related hospitalisation, or BD-related emergency room visits (Table 1). Moreover, CHIP was not an independent risk factor for poor clinical composite outcomes, including medical acceleration, surgery, hospitalisation, or emergency room visits, in patients with BD, in the Cox regression analysis (data not shown).

There was one patient with aplastic anaemia and two patients with myelodysplastic syndrome in our cohort. However, there was no CHIP mutation in the three patients.

## Discussion

To our knowledge, this is the first report to describe the development of CHIP in patients with BD and analysing the association between CHIP and inflammation in BD. We determined that the rate of CHIP in BD patients was not increased compared to the general population. The *DNMT3A* and *TET2* mutations were dominant in the control and BD groups. The CHIP rate significantly increased with age at the time of blood sampling. Furthermore, it was associated with a higher CRP level and ESR but negatively with albumin level at diagnosis. Finally, it was not associated with the clinical outcomes of BD.

Ageing, the strongest risk factor for CHIP development in patients with BD, is widely accepted and considered the biggest contributor to the accumulation of somatic mutations. Its intrinsic mechanisms include haematopoietic stem cell–induced age-dependent DNA damage, telomere attrition, and epigenetic dysregulation involving *DNMT3A* and *TET2* [17]. Interestingly, in addition to intrinsic mechanisms, various environmental stressors such as cytotoxic stress, autoimmunity, and infection contribute to CHIP emergence [18]. Here, we investigated the association between CHIP and elevated levels of inflammatory factors in patients with BD. Although the significance disappeared after the adjustment for age, ESR, CRP, and albumin levels were confirmed as highly correlated with CHIP in patients with BD by univariate logistic regression analysis. Inflammatory markers, including white blood cell and neutrophil counts, ESR, and CRP level, were not elevated in the general population with CHIP in a previous study [19]. However, CHIP carriers have higher CRP levels among patients with prior coronary artery disease [20]. In line with the results of previous studies, our results support the theory that CHIP is regulated by chronic inflammation, reflecting higher serum ESR and CRP and albumin levels in CHIP( +) BD patients. Regarding elevated platelet numbers in CHIP( +) patients, similar results were reported in a previous study (294,000/µL vs. 241,000/µL, *p* = 0.021) [21]. Regarding long-term clinical outcomes, CHIP could not predict medical acceleration, surgical requirement, BD-related hospitalisation, or BD-related emergency room visits. We appreciate the reviewer’s comment. There were some limitations in our study. First, there was a lack of validation using an independent cohort. Second, this study had low statistical power due to the small number of cases. Third, there is the possibility of other unknown variants associated with BD. We added these issues in the “Discussion” section.

## Conclusions

In summary, our study found that older age and degree of inflammation in BD might be associated with CHIP emergence.

## Data Availability

The datasets used and/or analysed during the current study are available from the corresponding author upon reasonable request.

## Abbreviations

ANCA: Anti-neutrophil cytoplasmic antibody
BD: Behçet’s disease
CHIP: Clonal haematopoiesis of indeterminate potential
CI: Confidence interval
CRP: C-reactive protein
ESR: Erythrocyte sedimentation rate
OR: Odds ratio
*TET2*: Ten eleven translocation-2
UC: Ulcerative colitis
VAF: Variant allele frequency
